# The Molecular Regulation of Carbon Sink Strength in Grapevine (*Vitis vinifera L*.)

**DOI:** 10.3389/fpls.2020.606918

**Published:** 2021-01-08

**Authors:** You-Mei Li, Charles Forney, Bhaskar Bondada, Feng Leng, Zhao-Sen Xie

**Affiliations:** ^1^College of Horticulture and Plant Protection, Yangzhou University, Yangzhou, China; ^2^Kentville Research and Development Centre, Agriculture and Agri-Food Canada, Kentville, NS, Canada; ^3^Wine Science Center, Washington State University, Richland, WA, United States

**Keywords:** grape, sink strength, sugar accumulation, sugar transporters, sugar-cleaving enzymes, hormones

## Abstract

Sink organs, the net receivers of resources from source tissues, provide food and energy for humans. Crops yield and quality are improved by increased sink strength and source activity, which are affected by many factors, including sugars and hormones. With the growing global population, it is necessary to increase photosynthesis into crop biomass and yield on a per plant basis by enhancing sink strength. Sugar translocation and accumulation are the major determinants of sink strength, so understanding molecular mechanisms and sugar allocation regulation are conducive to develop biotechnology to enhance sink strength. Grapevine (*Vitis vinifera L.*) is an excellent model to study the sink strength mechanism and regulation for perennial fruit crops, which export sucrose from leaves and accumulates high concentrations of hexoses in the vacuoles of fruit mesocarp cells. Here recent advances of this topic in grape are updated and discussed, including the molecular biology of sink strength, including sugar transportation and accumulation, the genes involved in sugar mobilization and their regulation of sugar and other regulators, and the effects of hormones on sink size and sink activity. Finally, a molecular basis model of the regulation of sugar accumulation in the grape is proposed.

## Introduction

With an increasing global population estimated to reach 9 billion people by 2050 (Nelson et al., 2012), a dramatic increase in agricultural productivity must occur. However, this increase has to be achieved without additional land for production and within the context of uncertain climatic shifts (Beddington, 2010). Thus, it is imperative to produce higher biomass and yield on a per plant basis to sustain the growing human population. In plants, the “source” refers to an organ with a net export of assimilates required for plant growth, such as carbon; whereas the “sink” is an organ that consumes or accumulates resources from the source (Doehlert, 1993). Photoassimilates are usually transported from sources to sinks as simple sugars, typically as sucrose (White et al., 2016). Sink strength determines photoassimilates importation and accumulation, thus controlling crop yield and affecting fruit quality. Clearly the phloem loading of sucrose at the source organ, transporting from source to sink, and unloading and metabolism in the sink organ are major processes affecting sink strength (Zhang et al., 2005). A complex signaling network encompassing sugars, hormones, and environmental factors determine sink strength by regulating sugar mobilization (Yu et al., 2015). Therefore, understanding sucrose delivery and accumulation regulation is vital to enabling new technological breakthroughs to improve crop yield and food quality.

Grape (*Vitis vinifera L.*) is a widely grown economic fruit species consumed as fresh fruit, dried raisins, and wine (Bondada et al., 2017; Imran et al., 2017; Khalil-Ur-Rehman et al., 2017; Wang W. et al., 2017). As in many crops, soluble sugar content is a primary component of yield and market value for grapes. Sucrose translocated from grape leaves is unloaded and metabolized in fruit (sink) and eventually accumulates as hexose (Lecourieux et al., 2014). Hexose accumulation not only provides carbon and energy for berry growth, but it also contributes to the sweetness and flavor of grapes. This review focuses on the mechanism related to the formation and regulation of sink strength in grape, including the molecular mechanism of sugar transport and accumulation, the genes involved in sugar allocation and its regulation, the genetic regulation of hormones and their role in regulating sugar transport, phloem unloading, and metabolism. This review also provides a premise for further research and development of new technologies to regulate photosynthetic assimilate partitioning in perennial fruit crops.

## Sink Strength

Sink strength has been defined as an organ’s competitive ability to import photoassimilates, which can be expressed as the product of sink size and sink activity (Ho, 1988). Sink size is the total biomass of the sink tissue (g), reflecting the cell number and size. Genetic factors and plant growth regulators regulate them during cell division (Bünger-Kibler and Bangerth, 1982; Geiger and Shieh, 1993). Sink activity refers to a particular resource’s specific uptake rate (mol g^–1^ s^–1^) (Geiger and Shieh, 1993). Three crucial physiological processes govern sink activity: phloem unloading and post-phloem transport of sugars in the sink cells; the absorption of the sink organ itself; and carbohydrate accumulation in sink organs (Roitsch, 1999). Also, external factors can modulate sink strength by increasing source strength (generally increasing source strength by influencing photosynthetic rate or increasing source capacity), regulating the loading and unloading of the assimilate in the phloem, and participating in the formation of sink size (cell number and size) and sink activity (sugar transport, phloem unloading, and accumulation in the sink organs). Studies on grapes have shown that the vital step controlling sugar accumulation is determined within the developing fruit rather than the source leaves’ ability to export photosynthetic products or the transport efficiency of the phloem pathway (Zhang et al., 2006; Xie et al., 2009). This key step occurs in the process of sugar entering berry cells after the sugar is unloaded from fruit phloem, which is called “post-sieve molecular transport” or “post-phloem transport” (Fisher and Oparka, 1996). It is the critical step that restricts the improvement of berry quality. Therefore, the external factors involved in regulating the sink activity, including sugar phloem unloading and sugar accumulation in the sink cells, could strongly affect sink strength in the grape.

## Molecular Biology of Sink Strength

Assimilate input and sucrose metabolism in sink organs directly affect the development and vitality of sink organs and determines sink strength (Chourey et al., 2012). Clarifying the molecular biology of sugar transport and accumulation is essential for the identification of the key enzyme/genes that control the distribution of assimilates from “source” to “sink” in perennials fruit crops.

### Sugar Transport and Accumulation in Grape

The grape berry exhibits a double sigmoidal growth pattern (Coombe, 1992). Its development can be divided into stage I and stage III, characterized by rapid growth, whereas stage II is a lag period. The first rapid growth phase that occurs after the fruit set is due to increased cell number and expansion of existing cells. Cell division in the flesh is almost complete during the first few weeks of development (Harris et al., 1968). Stage II, 7 to 10 weeks after flowering, is when little or no growth occurs. The second rapid growth phase occurs at the end of the lag phase. The transition from stage II to stage III is completed within 24 h, and is known as véraison, associated with a change in skin color, and marks the beginning of maturity (Coombe, 1992). Before véraison, the fruit is hard, green, acidic, and lacks sugar (<150 mM sugar content) and glucose to fructose ratio is about 2:1. During véraison, organic acid content begins to decrease, and soluble sugar concentration increases. About 20 days after the onset of véraison, the hexose concentration increases to around 1 M and the ratio of glucose to fructose is 1:1 (Findlay et al., 1987). Sucrose is exported from the source organ (leaf) and imported into berry flesh cells via two pathways: the symplastic pathway and the apoplastic pathway. Firstly, the sucrose, produced in the photosynthetic mesophyll cells (MC) of leaves, is loaded into the phloem sieve tubes through a symplastic-loading pathway or apoplastic-loading pathway. In the symplastic-loading pathway, sucrose is loaded into the sieve tubes through plasmodesmata between the sieve elements (SE)/companion cell (CC) complex and the surrounding parenchymal cells without specific carries (Turgeon and Wolf, 2009). In the apoplastic-loading pathway, sucrose is loaded and accumulated in the phloem by passing through the apoplast between the phloem parenchyma cells (PP) and the CC with specific transporters, which can be of the PLANT SUGAR WILL EVENTUALLY BE EXPORTED TRANSPORTER (SWEETs) family (Chen et al., 2012) and SUCROSE TRANSPORTERS (SUT1/SUC2) proteins coupled with a proton pump (H + − ATPase) (Gottwald et al., 2000). Then sucrose is transported in the sieve tubes via hydrostatic pressure. Following long-distance delivery, sucrose is unloaded from the phloem SE to the storage parenchyma cells through the symplastic-unloading pathway or the apoplastic-unloading pathway. In the latter case, sucrose is taken up into the parenchyma apoplasm by the members of the SUT1/SUC2 transporter family and is then hydrolyzed by cell-wall invertase (CWINV) or sucrose synthase into hexoses (Chen et al., 2017; Wan et al., 2018; Duan et al., 2020). Hexoses enter the parenchyma cells via specific monosaccharide transporters at the plasma membrane or the tonoplast level (Corelli Grappadelli et al., 2019). Alternatively, sucrose is imported into the vacuoles, where it is converted into hexoses by vacuolar invertase (VIN). These processes maintain a sucrose concentration gradient at the unloading site to maintain a high rate of unloading and hexoses accumulation (Lecourieux et al., 2014). Studies with grape have shown that the phloem unloading routes are changeable with grape sink development. A shift of phloem unloading from symplastic to apoplastic pathways was verified at or just before the onset of véraison, indicating the phloem unloading pathway changes in response to grape sink development (Zhang et al., 2006). It appears that sugar transporters and sugar metabolism-related enzymes are linked to a complex regulatory network that determines the fruit’s sugar accumulation.

### Sugar Transporters and Sugar-Cleaving Enzymes in Grape

Four sucrose transporters (SUT/SUC) were identified in the grape genome (Table 1), three of them were cloned from Shiraz and Cabernet Sauvignon berries (VvSUC11; AF021808, also identified as VvSUT1 AF182445; VvSUC12 AF021809; and VvSUC27 AF021810) and the sucrose transport activity was characterized by heterologous expression in *Saccharomyces cerevisiae* (Afoufa-Bastien et al., 2010). VvSUC11 belongs to the SUT4 subfamily, including AtSUC4. VvSUC12 contains two structural characteristics specific to the SUT2/SUC3 subfamily, which includes AtSUC3. VvSUC27 is a SUT located at the plasma membrane and belongs to the dicot-specific SUT1 subfamily, which includes the remaining AtSUCs (Cai et al., 2017). Overexpression of *VvSUCs* (*VvSUC11* or *VvSUC12*, or *VvSUC27*) in both tobacco and Arabidopsis conferred more rapid development, higher yield and enhanced abiotic stress resistance (Cai et al., 2017, 2020). Similarly, Cai et al. (2019) reported *SUTs* in ‘Zuoshan-1’ (*Vitis vinifera*) responded to various stress stimuli and subsequently promoted sucrose metabolism and hormone synthesis.

**TABLE 1:** The summary information of sugar transporters identified in grape.

**Type**	**Genes**	**Family members**	**Substrate**	**Location**	**Cloned members in grape**	**Function of cloned members**	**References**
Disaccharide transporter	VvSUT/VvSUC	4	Sucrose	Plasma membrane	VvSUC11, VvSUC12, VvSUC27	Sucrose transport; Promote development; Enhance abiotic stress	Afoufa-Bastien et al., 2010; Cai et al., 2017, 2019, 2020
Monosaccharide transporter	VvHT (sub I) VvTMT (sub II) VvPMT (sub III) ERD6-like (sub IV) VvVGT (sub V) VvINT (sub VI) VvGlcT (sub VII)	59	Hexose, Polyol, Inositol	Plasma membrane or tonoplast	VvHT1, VvHT4, VvHT5, VvTMT1, VvHT2, VvHT6	No detail in grape	Fillion et al., 1999; Vignault et al., 2005; Conde et al., 2006; Hayes et al., 2007; Agasse et al., 2009; Afoufa-Bastien et al., 2010; Zeng et al., 2011
Sugar Uniporter	VvSWEET	17	Sucrose, Glucose, Fructose	Plasma membrane	VvSWEET4, VvSWEET7, VvSWEET10	Broad spectrum of sugar transport; Enhance biotic stress	Chong et al., 2014; Breia et al., 2019; Meteier et al., 2019; Zhang et al., 2019

Monosaccharide transporter genes are present in 59 loci in grapevine, which can be grouped into seven subfamilies, including 20 hexose transporters (VvHT; subfamily I), three tonoplast monosaccharide transporters (VvTMT; subfamily II), five polyol/monosaccharide transporters (VvPMT; subfamily III), ERD6-like Transporters (subfamily IV), two vacuolar glucose transporters (VvVGT; subfamily V), 3 inositol transporters (VvINT; subfamily VI) and other four monosaccharide transporters (VvpGlcT/VvSGB1; subfamily VII) (Table 1; Afoufa-Bastien et al., 2010). Seven full-length cDNAs encoding monosaccharide transporter, named VvHT1–VvHT6/VvTMT2, VvTMT1 (VvHT1 AJ001061; VvHT2 AY663846; VvHT3 AY538259 and AY854146; VvHT4 AY538260; VvHT5 AY538261; VvHT6 AY861386, DQ017393) have been isolated from various cultivars such as Pinot noir, Ugni blanc, Chardonnay, Cabernet Sauvignon, Syrah and Riesling (Fillion et al., 1999; Vignault et al., 2005; Hayes et al., 2007; Zeng et al., 2011). Three of them (VvHT1, VvHT4, and VvHT5) belong to plasma membrane hexose transporters. Their plasma membrane localization has been verified by immunofluorescence, immunolabeling, and GFP fusion proteins. They all facilitated glucose uptake, VvHT1 has a higher affinity for glucose than VvHT4 and VvHT5 and displays broad substrate specificity, being able to recognize both d-glucose and d-fructose. On the contrary, VvHT3 is not able to transport any of the tested radiolabeled sugars in the deficient yeast model (Vignault et al., 2005; Conde et al., 2006; Hayes et al., 2007). VvHT2 and VvHT6/VvTMT2 seem to be localized in the tonoplast and VvHT6/VvTMT2 has high sequence similarity to the previously described tonoplast monosaccharide transporter of *Arabidopsis thaliana* AtTMT2 (Agasse et al., 2009; Afoufa-Bastien et al., 2010). Induction of VvTMT1-GFP fusion protein expression in transgenic yeast revealed its tonoplast localization. Glucose/other monosaccharide -uptake activities of VvTMT1 have been detected by heterologous expression in the *hxt*-null mutant yeast (Zeng et al., 2011). Recent function analysis indicated *PbTMT4* regulates the accumulation of sugars in the vacuole and it is a strong contributor to fructose, glucose, and sucrose accumulation in frutescence of pears (*Pyrus bretschneideri*) (Cheng et al., 2018). Transient silencing of one *Prunus persica* tonoplast sugar transporter (*PpTST1*) significantly inhibited sugar accumulation in peach fruit (Peng et al., 2020). For ripe grapes, a very high concentration of glucose and fructose (∼1 M each) accumulates in the vacuole of flesh cells (Fontes et al., 2011). Clearly, monosaccharide transporters play important roles in vacuolar accumulation of hexose in grape berry. To date, the function of these transporters has not been studied in detail, though different locations and affinity for monosaccharide of these monosaccharide transporters showed diversified functions in sugar transport process.

SWEETs is a family of a plasma membrane-localized sugar uniporters that have been found in recent years. *Arabidopsis thaliana* AtSWEET1 was the first identified plant SWEET transporter and responsible for glucose unidirectional transport in different systems (Chen et al., 2010). AtSWEET11, AtSWEET12, and AtSWEET17 were subsequently characterized to export sucrose out of the phloem parenchyma cells or fructose efflux from vacuole (Chen et al., 2012; Chardon et al., 2013). Seventeen SWEET homologes were identified in the grape genome (Table 1), VvSWEET4 is characterized as a glucose transporter localized in the plasma membrane (Chong et al., 2014). Overexpression of the *VvSWEET4* in grapevine hairy roots increases sugar transportation and content and enhances resistance to soilborne pathogens (Meteier et al., 2019). Similarly, VvSWEET7 is a mono- and disaccharide transporter and its expression is up-regulated in response to *Botrytis cinerea* infection in grapes (Breia et al., 2019). VvSWEET10 has a broad spectrum of sugar transport functions. Overexpression of *VvSWEET10* in grapevine calli and tomatoes increased the glucose, fructose, and total sugar levels significantly (Zhang et al., 2019). *VvSWEET15* was highly expressed in three grape cultivars’ berries and obviously positively correlated with berry hexose content during ripening (Ren et al., 2020). In summary, these findings demonstrated the role of *VvSWEETs* in sugar accumulation and biotic stress response.

**FIGURE 1:**
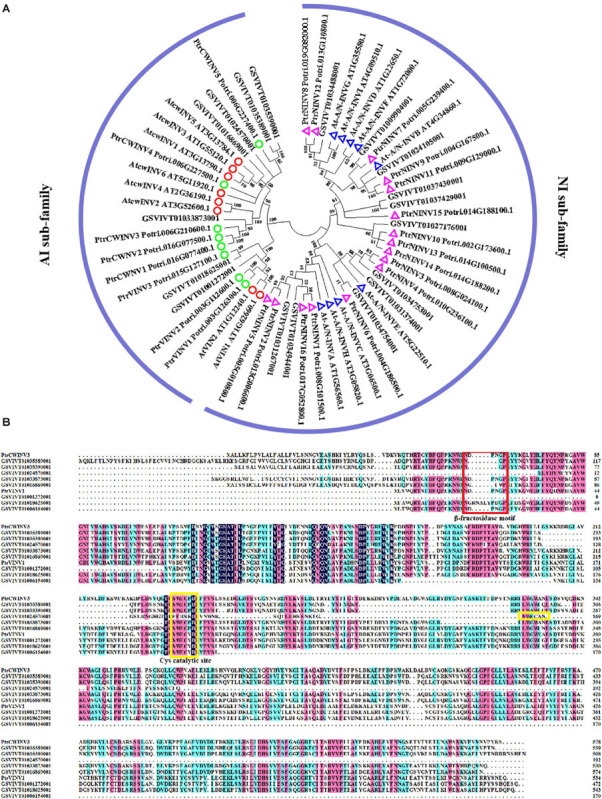
**(A)** Phylogenetic tree of invertase genes from different plants. Amino acid sequences from Arabidopsis (tair database), populus (Chen et al., 2015) and grapevine were used to construct the unrooted phylogenetic tree by MEGA v. 6.0 (Tamura et al., 2013) with the neighbor-joining (N-J) method, p-distance, pairwise deletion method, and a bootstrap test with 1,000 replicates. The red and green circles indicate vacuolar acidic invertases and cell wall acidic invertases of Arabidposis and populus, respectively. Blue and purple triangles indicate alkaline/neutral invertase of Arabidposis and populus, respectively. **(B)** Multiple alignment of the acid invertase sub-family in grape with two known populus vacuolar acidic invertase (PtrVINV1) and cell wall acidic invertase (PtrCWINV3) as references. The red box shows β-fructosidase motif (DPNGP), the yellow box shows the cys catalytic site (MWEC-P/V-D).

After importation into sink parenchyma cells, sucrose is rapidly taken into the vacuole for storage or degraded into hexoses for a wide range of metabolism in order to maintain the gradients in the sink cells, thus maintaining phloem unloading (Lecourieux et al., 2014). Sucrose-cleaving enzymes such as sucrose synthase (SuSy) and invertase are mainly responsible for sucrose metabolism in sink cells (Chen et al., 2017; Wan et al., 2018). Stein and Granot (2019) reviewed SuSy is the only Suc-metabolizing enzyme that can catalyze both the synthesis of sucrose from fructose and uridine diphosphate glucose (UDP-G) and the cleavage of sucrose, in the presence of UDP or other nucleotide phosphates (especially ADP), to fructose and UDP-G or adenosine diphosphate glucose (ADP-G). Subcellular localization of SuSy is detected both in cytosolic fractions or/and plasma-membrane in several plants. SuSy contributes to sucrose hydrolysis mainly in the cytosol and it responds to sink strength in different plants (Braun et al., 2014). Five SuSy genes (VvSS1-5) have been identified in the grape genome (Zhu et al., 2017). *VvSS3* was reported to co-express with *VvSWEET15* to regulate sucrose hydrolysis and transport, leading to increase hexose accumulation in grapes (Ren et al., 2020). Invertase hydrolyzes sucrose irreversibly into glucose and fructose and has a crucial function in establishing and maintaining sink metabolism, and hence considered to be a central molecular sink strength determinant (Koch, 2004). Three types of invertase isoenzymes are distinguished based on their solubility, subcellular localization, pH optima, and isoelectric point: (1) Soluble acid invertase (VIN) is located primarily in vacuoles; (2) Cell wall-bound invertase (CWINV) binds to cell walls; (3) Soluble alkaline or Neutral invertase (A/N-INV) is present in the cytoplasm and has a low activity in plant tissues (Sturm, 1996). Invertases are also divided into Acid Invertase (AI) and Neutral Invertase (NI) according to the optimal pH required by the reaction (Avigad, 1982). To our knowledge, nine neutral/alkaline invertase (NI) genes have been identified in the 8.4X grape genome (Nonis et al., 2008). But genes encoding AI have not been identified so far. We conducted a BLASTP search against the 12X grape genome database (Grapevine Genome Browser. Available online^1^) using known protein sequences of invertase genes from Arabidopsis as queries (tair datebase. Available online^2^). After manually removing redundant sequences and verifying existence of the core domains with the Conserved Domains Database (CDD. Available online^3^), 19 putative invertase genes were identified in the grape genome (Supplementary File 1). The phylogenetic analysis revealed that 11 were grouped into the neutral/alkaline invertase sub-family, and eight were from the acid invertase sub-family, of which there were three vacuolar acidic invertases (VIN) and five cell wall acidic invertases (CWINV) (Figure 1A). Multiple alignments of acid invertase sub-family (Figure 1B) revealed that the only one CWINV (GSVIVT01033873001) and one VIN (GSVIVT01006154001) contain the DPNGD domains, which were well-conserved in this family and are essential for β-fructosidase catalytic activity (Sturm and Chrispeels, 1990). The other six genes contain the variable NDPNG motif. The conservative Cys catalytic site (MWEC-P/V-D) (Chen et al., 2015) was found at the C-terminal of all acid invertase members, the exception being one VIN gene (GSVIVT01024570001) with ‘MWECAN’ motif, a proline (P) residue was present in MWEC-P/V-D motif of CWINV sequences, whereas a valine (V) was at the same position of VIN sequences. This finding is consistent with the previous study (Goetz and Roitsch, 1999). The variation of NDPNG motif among acid invertase genes may indicate their specific function in grape. Several *AI* genes have been functionally validated in sink strength through their involvement in sucrose metabolism in some species (Liu et al., 2016; Xu et al., 2019; Deng X. et al., 2020). In grape, an increase in the expression and activity of CWINV was concomitant with a rise in apoplastic sugar concentration and osmotic pressure (Zhang et al., 2006; Xie et al., 2009). While some studies reported invertase and hexose transporters are unnecessary for sugar differential accumulation among cultivars (Ren et al., 2020), others found that it was mainly due to acid invertase activity (Guan, 2011). However, no functional identification is focused on the member of VvSSs or invertase family in grape so far. Therefore, a transgenic functional characterization experiment would help explore the specific role of each VvSSs or invertase genes in sugar accumulation in grape berry.

### The Regulation of Sugar Transporters and Sugar-Cleaving Enzymes

The current investigations opine that the plant sugar transporters and sugar-cleaving enzymes under the control of their substrate either at the transcriptional or post-transcriptional levels. Exogenous sucrose feeding controls the transcription level of *SUC* and SUC protein amount in sugar beet (Vaughn et al., 2002). Glucose could regulate the expression of monosaccharide transporters (*VvHT3*, *VvHT4*, *VvHT5*, and *VvHT6*) by inducing *VvSK1* transcription, a Glycogen Synthase Kinase3 protein kinase that modulates sugar uptake and accumulation in grape (Lecourieux et al., 2010). Several studies reported that extracellular invertases are induced by sugars (Roitsch et al., 1995; Tymowska-Lalanne and Kreis, 1998). The expression of *CWINV* and *SuSy* was repressed by glucose, and the intensity of repression depended on glucose concentration and incubation time. Hexokinase (HXK, EC 2.7.1.1) involved in hexose phosphorylation, plays a crucial role in sugar sensing and signaling. It has been found HXK activity had an inverse relationship with the endogenous glucose or fructose levels during grape development. The phosphorylation of hexoses by HXK was an essential component in the glucose-dependent *VvCWINV* and *VvSuSy* expression (Wang et al., 2014). Additionally, glucose could repress *VvHT1* transcription via an HXK-dependent pathway. While inducing the reduction of VvHT1 protein in the plasma membrane by a HXK-independent post-transcriptional regulation (Conde et al., 2006). Thus, it is clear that sugar could play a regulatory role in sink activity by regulating its transport carrier or metabolic enzymes.

Except for sugar’s regulatory role, little is known about protein/regulators that control sugar transporters and metabolic enzymes in the grapevine. In Arabidopsis, identification of SUC2-interaction partners indicated that SUC2 activity is controlled by its protein turnover rate and phosphorylation state. UBIQUITIN-CONJUGATING ENZYME 34 (UBC34) is responsible for triggering the turnover of SUC2 in a light-dependent manner. WALL-ASSOCIATED KINASE LIKE 8 (WAKL8) functions on the phosphorylation of SUC2 for increasing transport activity (Xu et al., 2020). In cotton (*Gossypium hirsutum*), CBL-interacting protein kinase GhCIPK6 is recruited to the tonoplast by Calcineurin B-like protein GhCBL2 and interacts with the tonoplast-localized sugar transporter GhTST2 to regulates plant sugar homeostasis, in particular glucose homeostasis (Deng J. et al., 2020). HpWRKY3, a WRKY transcription factor in pitaya (*Hylocereus*), was identified as the putative binding protein of the *HpINV2* and *HpSuSy1* promoters and could activate the expression of *HpINV2* and *HpSuSy1*. It is proposed that HpWRKY3 is involved in sugar accumulation by inducing the sucrose metabolic genes in pitaya fruit (Wei et al., 2019). Similarly, Li et al. (2020) found that PuWRKY31 bound to the *PuSWEET15* promoter and induced its transcription to promote sugar accumulation in Ussurian pear (*Pyrus ussuriensis*).

## The Effect of Hormone on Sink Strength

Grape production benefits from plant growth regulators as long as they are applied at the recommended dose and frequency, and pre-harvest intervals are strictly followed (Ugare et al., 2013). For instance, CPPU [forchlorfenuron,N-(2-Chloro-4pyridinyl-N-phenylurea)], a synthetic cytokinin-like plant regulator, promotes berry set and increases berry size (Reynolds et al., 1992). Application of gibberellic acid (GA) is a routine practice to increase berry size or induce seedlessness in table grapes (Weaver and MacCune, 1961); Other plant hormones such as Abscisic acid (ABA), auxin, and brassinosteroids (BR) also play important roles in the formation and maintenance of fruit sink strength.

### Cytokinin

A study that tested nine plant hormones found that only 6-benzylaminopurine (6-BA, artificial agent for cytokinin) and ABA stimulated unloading photosynthetic products in bean seeds, which occurred within 10–12 min after 6-BA or ABA treatment (Clifford et al., 1986). Several studies supported that cytokinin may regulate rate-limiting steps in nutrient utilization and distribution. For instance, radioactive nutrients were preferentially transferred and accumulated in cytokinin-treated areas (Mothes and Engelbrecht, 1962). Localized overexpression of the *ISOPENTENYLTRANSFERASE* (*IPT*, the enzyme that mediates the rate-limiting step in cytokinin synthesis) caused local sink enhancement (Guivarc’h et al., 2002). The formation of green areas on senescing leaves suggested an alteration of the sink-source relation by cytokinin (Engelbrecht et al., 1969; Body et al., 2013). A later study found photosynthetic capacity and soluble sugar content were not significantly affected in the source organs (leaves) of a cytokinin-deletion mutant, however, soluble sugar content, invertase activity and ATP content in sink organs (shoot tip) were obviously reduced (Werner et al., 2008). Recent research reported phytochromobilin deficiency impairs sugar metabolism through the regulation of cytokinin and auxin signaling in tomato fruit (Bianchetti et al., 2017). Additionally, light can weaken the sink activity and inhibit axillary bud germination by downregulating cytokinin signals in rose. Under dark conditions, 6-BA treatment rapidly activated the key components of sink strength, including vacuolar acid invertase, sucrose transporter and sucrose synthase, and promoted axillary bud growth (Roman et al., 2016). In summary, these findings support the role of cytokinin in regulating sink strength, and suggest that localized cytokinin production could create a localized sink and result in a new source-sink relationship, thereby modulating nutrient mobilization.

Several studies have related the availability of cytokinin to plant sink strength under abiotic stress in recent years. For instance, the exogenous application of cytokinin (KIN) to developing tomato fruit or the increase in endogenous cytokinins (tZ) under salinity partially restored sink activity and fruit growth, and also activated most sugar metabolic enzymes (Albacete et al., 2014). Delay of senescence in *IPT* transgenic tobacco (*Nicotiana tabacum*) plants correlated with elevated CWINV activity. Localized induction of a *CWINV* under control of a chemically inducible promoter resulted in an ectopic delay of senescence, resembling the naturally occurring green areas on senescing leaves, these results established a causal relationship between cytokinin and extracellular invertase for delaying senescence (Balibrea Lara et al., 2004). P(SARK): IPT transgenic rice plants, expressing *IPT* driven by P(SARK), a maturation- and stress-induced promoter, had enhanced drought tolerance and higher grain yield with improved quality (nutrients and starch content). This phenomenon indicated that stress-induced cytokinin synthesis in the P(SARK):IPT plants modified source/sink relationships and promoted sink strength through a cytokinin-dependent coordinated regulation of carbon and nitrogen metabolism that allowed plant adaptation and survival under water stress (Reguera et al., 2013). Results in many crops that overexpress *IPT* under the control of P(SARK) have demonstrated a delay in leaf senescence and improved crop growth and yield under different stress conditions (Décima Oneto et al., 2016; Joshi et al., 2019). Therefore, it appears that cytokinin plays a role in making up the deficiencies caused by stress in plant. Cytokinin may function in two ways when plants are under abiotic stress. Cytokinin could delay the stress response or senescence of leaves (thus maintaining source activity), resulting in the production and/or export of more assimilates for sink organs’ growth. On the other hand, cytokinin could expand the sink capacity by increasing cell proliferation or maintaining the sink activity by regulating sucrolytic enzymes to acquire more photo-assimilates.

Forchlorfenuron,N-(2-Chloro-4pyridinyl-N-phenylurea) is widely used for fruit size enhancement in table grape production (Reynolds et al., 1992; Wang W. et al., 2017). Hence, it may be suggested that CPPU treatment is conducive to forming the sink capacity at early berry development. Indeed, endogenous cytokinin levels are critically involved in the regulation of early fruit growth through the regulation of cell division by D-type cyclin expression (Baldet et al., 2006). Similar results were also observed with barley (Hordeum vulgare L.), wherein the effects of foliar application of 6-BA resulted in increased sink size soon after anthesis and increased sink demand was met by current photosynthesis of organs (Hosseini et al., 2008). However, the application of CPPU had some adverse effects on grapes, including inhibiting anthocyanin and sugar accumulation (Khalil-Ur-Rehman et al., 2017). Moreover, the endogenous cytokinin zeatin levels are high early in the flesh of immature berries but decrease rapidly at the time of véraison when sugar and anthocyanin start to accumulate (Zhang et al., 2003). On the other hand, some studies found a rapid increase in isopentenyl adenine (ip) at véraison and remained at elevated levels throughout grape ripening (Böttcher et al., 2013, 2015). To date, studies have not given any clear indications for possible functions of endogenous cytokinin on the sugar accumulation in grapes. Therefore, why CPPU caused decreased sugar accumulation in grapes is still unknown. This adverse effect may be related to two main reasons. One is the ability of cytokinin to establish local metabolic sink capacity. Under normal growth conditions, exogenous cytokinin application could reconstruct other competitive sink organs and break the original balance between the source (leaf) and sink (berry), resulting in reduced accumulation of sugar in fruit. The second reason is that cytokinin prefers to enhance sink size rather than to sink activity. Application of CPPU could trigger fruit growth and delay fruit maturity, therefore not conducive to initiating fruit ripening and sugar accumulation. Future studies should focus on fine-tuning the regulation of cytokinin concentrations and the tissue-specific responses during grape development.

### Gibberillic Acid

A two-step GA application has usually been employed before anthesis and again after anthesis; the former for inducing seedlessness and the later for berry enlargement (Dass and Randhawa, 1968; Wang X. et al., 2017). GA_3_ is thought to increase fruit size by triggering cell division and expansion in many fruit crops (Zhang and Whiting, 2011). Recently, transcriptome analysis revealed that cell-wall relaxation could be the main process in exogenous GA3-triggered berry enlargement at the early stage of grape fruit development (Chai et al., 2014). GA_3_ application at the 3–4 mm berry stage increased grape size, and the top five genes upregulated by GA_3_ are related to cell wall formation in berries (Upadhyay et al., 2018). Similarly, proteome analysis of the berry-sizing effect of GA_3_ on seedless grape shows cytoskeleton and cell-wall modification proteins are up-regulated in the stages II and III of berry development (Wang et al., 2012). Hence, post-bloom GA_3_ application induced berry expansion through modifying cell wall components. Recent studies reported GA could regulate genes of the lignin biosynthesis (Garcia-Rojas et al., 2018), cell wall metabolism, xylem development, phenylpropanoids, and the cell cycle (Meneses et al., 2020), generating changes in cell wall composition that caused an increase in berry drop. These studies provide some clues to explore further the mechanism underlying the effects of GA on the fruit cell cycle and expansion affected by GA.

Sucrose, glucose, and fructose in grapes’ suspension cell cultures significantly increased after GA_3_ treatment (Zhang et al., 2014). These findings, coupled with previous studies, corroborate that GA_3_ can hasten the accumulation of hexoses within the expanding cells (Kordel and Kutschera, 2000). GA_3_ could relieve the repression of glucose on *CWINV* and *SuSy1* expression by regulating the *HXK* gene expression, thereby further regulating intracellular glucose metabolism to maintain normal cell growth in grape (Zhang et al., 2014). Similarly, a significant increase in sink demand and larger fruit size caused by applied GA was shown in Japanese pear. This enhanced sink strength was closely correlated with increased activities of sugar metabolizing enzymes induced by GA application during rapid fruit growth (Zhang et al., 2007). The expression of the Rosa hybrida vacuolar invertase 1 gene (*RhVI1*) was controlled by sugar/light and gibberellin/light synergistically. Further study demonstrated that the 127 bp RhVI1 promoter fragment located between −595 and −468 bp was critical for this synergism (Rabot et al., 2014). Therefore, GA_3_ functions as a regulator of sugar accumulation in the plant; however, the precise mechanisms involved remain elusive in many plants, including grape.

### Abscisic Acid

Extensive studies showed that ABA enhanced sugar accumulation in crop sink organs (Kobashi et al., 2001; Kuhn et al., 2013). In Malbec grapevines treated with ABA, increased accumulation of glucose and fructose in berries was correlated with enhanced *VvHT2* and *VvHT6* gene expression and increased phloem area and sucrose content in leaves (Murcia et al., 2016). When Hayes et al. (2010) studied the pathogen-induced regulators of carbohydrate sink strength, they found powdery and downy mildew infections induced *VvHT5* activity in coordination with *VvCWINV* in grape leaves and this was controlled through ABA. These findings indicated ABA regulated *VvCWINV* and *VvHT5* expression during the transition from source to sink in response to infection by biotrophic pathogens (Hayes et al., 2010). Some cross-mediation among ABA signaling and sugar metabolism and accumulation have been identified in different species. MdAREB2, an ABA-responsive transcription factor in apple (*Malus domestica*), directly activated the amylase and the sugar transporter gene *MdSUT2* to promote soluble sugar accumulation (Ma et al., 2017). The grape ASR (ABA, stress, ripening) protein VvMSA, a protein induced by sucrose, stress and ABA during fruit development, combines to a 160 bp interval of *VvHT1* promoter to regulate sugar movement and accumulation via regulating the expression of *VvHT1* (Carrari et al., 2004). A *VvMSA* transcriptional regulation model at the convergence of ABA, glucose, hexokinase1, and SnRK1 was proposed. In short, *VvMSA* expression is inhibited by hexokinase1 and stimulated by ABA at high glucose levels, whereas the inhibition by hexokinase1 is released at low glucose levels in grape protoplasts (Saumonneau et al., 2012; Dominguez and Carrari, 2015). ABSCISIC ACID-INSENSITIVE4 (ABI4) is a member of the AP2/ERF family. Its expression is induced in the presence of low glucose concentrations (Cho et al., 2010) and high glucose concentrations and ABA (Arroyo et al., 2003; Zheng et al., 2019). An ATX5-HY1-ABI4 regulatory module governing the glucose response was identified recently. More specifically, trithorax-group Protein ARABIDOPSIS TRITHORAX5 (ATX5) directly regulates the transcription of *HY1* by trimethylating H3 lysine 4 of the Arabidopsis *Heme Oxygenase1* (HY1) locus. Glucose signaling suppresses ATX5 activity and subsequently reduces the H3K4me3 levels at the HY1 locus, thereby leading to the increased expression of *ABI4* (Liu et al., 2018). ABA not only could activate both the *VIN* and *CWINV* during grape berry development through enhancing their activities and amounts (Pan et al., 2010), but also could block the inhibitory effect of glucose on the expression of *SuSy* and *CWINV* in grapes. This inhibition was linked with the glucose sensor HKX1 (Wang X. Q. et al., 2017). Therefore, HXK may be another potential cross-mediator between ABA and glucose signaling to control sugar accumulation.

### Auxin

Fruit growth usually begins with cell division, continues with cell division and expansion, and only ends with cell expansion. The SAUR19 subfamily, one of the auxin signaling components, has been shown to function as a positive effector of cell expansion in Arabidposis (Spartz et al., 2012). The cell elongation bHLH protein (VvCEB1) was identified and characterized to control cell expansion in grape. VvCEB1opt-overexpressing lines significantly elevated auxin content and increased the number of lateral leaf primordia within meristems relative to control, demonstrating that cell expansion and organ number proliferation were likely an auxin-mediated process (Lim et al., 2018). Overexpression of *VvCEB1* in grape embryos resulted in either activation or repression of the *VvIAA* family members’ expression while inducing the expression of *VvSAUR* and *VvGH3* genes. These disparities in the expression levels of auxin-regulated genes may reflect their complex regulation of cell expansion (Nicolas et al., 2013). Combined physiological, transcriptome and cis-regulatory element analyses in grapes suggest that fruit size is associated with changes in the berry’s ripening physiology, where large berries approach ripening faster. Compared with large berries, auxin levels are high in small berries, accompanied with upregulation of transcripts encoding TAR4 and YUC (Wong et al., 2016). It may be related to inhibition of cell expansion and fruit ripening by auxin. A recent study reported that auxin slowed down the onset of grape berry ripening by delaying cell expansion (Dal Santo et al., 2020). Taken together, these data provide insight into the link between auxin signaling and cell expansion in grapes, which may affect the sink capacity and sink demand in subsequent berry development.

Additionally, Böttcher et al. (2012) reported that the initiation of sugar accumulation was delayed. The sugar accumulation rate was lower in NAA-treated grape berries, resulting in a 15-day delay in harvest. Antisense suppression of Sl4RF4, an auxin response factor (ARF) gene in tomato (*Solanum lycopersicum*), resulted in higher starch content in developing fruits correlating with the up-regulation of genes and enzyme activities involved in starch biosynthesis. This phenomenon suggested the involvement of ARFs in the control of sugar content (Sagar et al., 2013). Altogether, the findings indicated that auxin delayed the accumulation of sugar content during fruit development.

### Brassinosteroids

Application of exogenous BR (24-epibrassinolide; EBR) increased soluble sugar content in Cabernet Sauvignon berries accompanied by increased activities of invertases and sucrose synthase. These increases coincided with the upregulation of transcription levels of *VvCWIN*, *VvHT3*, *4*, *5*, *6*, and *VvSUC12*, *27* during véraison (Xu et al., 2015). Vergara et al. (2018) also found that BR analogs increased soluble solids and anthocyanins in ‘Redglobe’ grapes. Together, these data suggested that BR positively controls sugar accumulation in grapes during véraison. In the review of Kuhn (2016), it was concluded that sugar transporter SUT2 and BR signaling cross-talk regulated plant immunity. Recent studies found that the phenotypic effects of *SlSUT2* silencing in tomato could partially be rescued by EBR treatment, demonstrating that SlSUT2 interconnects sucrose partitioning with brassinosteroid signaling (Hansch et al., 2020). These studies could provide guidance for further research into the involvement of BR in the regulation of sugar transport and accumulation in grapes.

**FIGURE 2:**
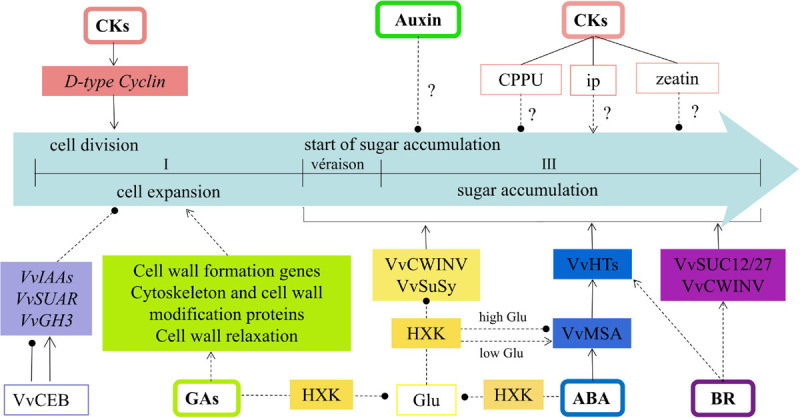
The regulation model of hormones on sink strength reported in grape. Symbol ‘?’ means no clear relationship between hormones and sink activity (sugar accumulation), → stands for promotion, —∙ stands for inhibition. Solid line means direct regulation relationship, dotted line means indirect regulation relationship or no further study for direct regulation.

To summarize, the regulation of hormones on sink strength reported in grape berry was constructed to a regulatory network in the present review (Figure 2). Cytokinin could facilitate sink size formation by activating D-type cyclin gene expression to promote cell division. However, exogenous cytokinin (CPPU) is not conducive to sugar accumulation in grape berry. The level of endogenous ip increased rapidly at véraison when sugar and anthocyanin accumulate. It remained at elevated levels throughout grape ripening, whereas zeatin decreased rapidly, reaching low levels at around véraison. However, no studies confirm the repression of zeatin or the promotion of ip on sugar accumulation. Auxin was found to inhibit cell expansion in grape berry. However, the differential response of auxin-related genes to the cell elongation bHLH protein (VvCEB1) suggests their complex cell expansion regulation. Recent reports suggest auxin could restrain the sink activity via delaying the initiation of sugar accumulation. GA is widely used to expand berry size at an early stage of grape development; this expansion could be involved in the regulation of cell wall formation genes, cytoskeleton and cell-wall modification proteins, and cell-wall relaxation process by GA. GA is also reported to contribute to sugar accumulation by alleviating glucose inhibition on the expression of *VvCWINV/VvSuSy*. The same regulation was found in ABA; both alleviate glucose’s repression on the *VvCWINV/VvSuSy* expression by an HXK dependent pathway. Additionally, VvMSA could respond to ABA to directly promote hexose transporter genes and promote sugar accumulation. BR could upregulate expression of *VvCWIN*, *VvHT3*, *4*, *5*, *6*, and *VvSUC12*, *27*, and facilitate to sugar accumulation during the véraison stage of grape development. More work is needed to fill the gaps in this network.

## Conclusion and Prospects

**FIGURE 3:**
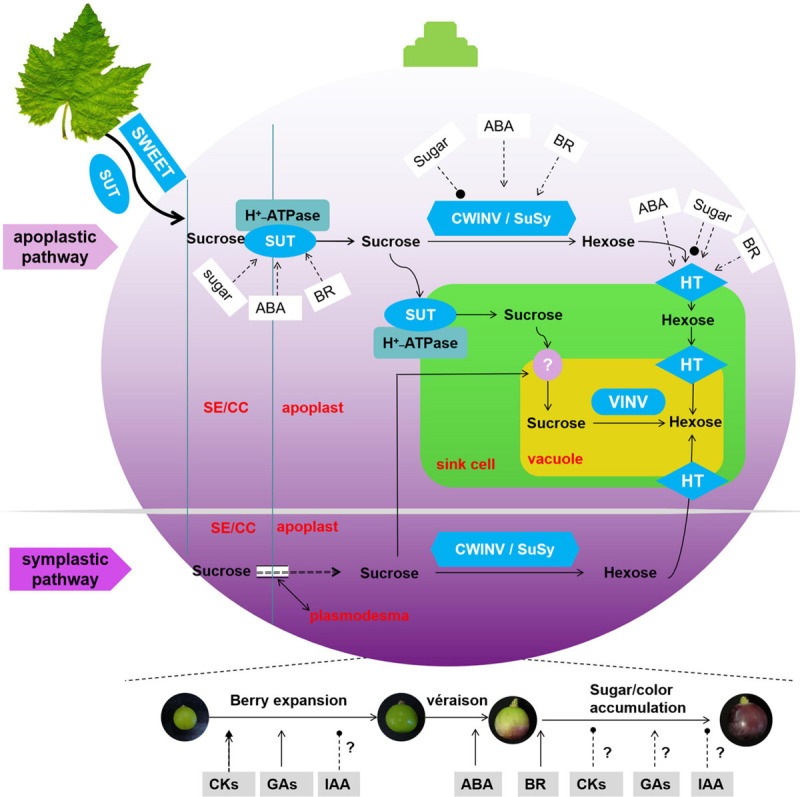
Model of the molecular basis for the progress and regulation of sugar accumulation in grape berry. Symbol ‘?’ means no clear relationship between hormones and sink size (berry expansion) and sink activity (sugar accumulation), → in blue stands for promotion, —∙ in blue stands for inhibition.

The present status of our knowledge on the regulation of sink strength in grapevine is summarized in Figure 3. The pattern of sugar accumulation during berry development is clearly obtained from previous studies. Grapevine genome sequencing has made a valuable contribution to identify additional genes encoding sugar transporters and metabolic enzymes. These genes are involved in the complex molecular biology of sugar transport and accumulation. However, most research showed only the average levels of the gene expression and enzyme activity for whole berries or grape heterotrophic suspension-cultured cells system. In contrast, sugar concentration is different in the flesh tissue (low hexose concentration near the brush, high at the stylar end). Active uptake of d-glucose in the flesh is only detected at a specific stage (véraison), while it can be detected at both pre- and post-véraison stage in isolated skin pieces (Coombe, 1992). So advanced studies are needed to determine the precise function of these genes in specific phloem locations of the grapevine so that the precise tracking of sugar allocation is revealed. Moreover, the proteins or transcription factors that modulate sugar transporters and metabolic enzymes could be further explored at the transcription or post-transcription levels. The potential regulation of the sugar transporters and metabolic enzymes would provide practical benefits, maximizing crop yield, and improving food quality.

Additionally, the role of various hormones in the regulation of sink size and activity is currently in progress in fruits (Figure 3). Although it is well established that ABA and BR promote sugar accumulation in grape, the mechanisms of these promotions remain to be elucidated. Cytokinin and GA are well known to be involved in berry expansion, promoting sink capacity, but no clear relationship has been established between sugar accumulation and endognous/exogenous cytokinin in grape. Several studies have contributed to the regulation of auxin regarding sink size and activity; however, the disparities in the expression levels of auxin-related genes reflect their complex regulation in cell expansion and sugar accumulation. To use hormones more effectively in grape production, we need a finely tuned regulation of hormone concentration and type that controls cell expansion/sugar allocation at given tissue locations during grape berry development.

## Author Contributions

Y-ML and Z-SX jointly wrote and revised the manuscript. CF and BB revised and rewrote sections of the manuscript. FL involved in revising the manuscript. All authors contributed to the article and approved the submitted version.

## Conflict of Interest

The authors declare that the research was conducted in the absence of any commercial or financial relationships that could be construed as a potential conflict of interest.

## Footnotes

1 http://www.genoscope.cns.fr/externe/GenomeBrowser/Vitis/

2 https://www.arabidopsis.org/servlets/Search?type=general&action=new_search

3 http://www.ncbi.nlm.nih.gov/cdd

## Abbreviations

At: *Arabidopsis thaliana*
Vv: *Vitis vinifera L*
Ptr: *Populus trichocarpa*
SE: sieve elements
CC: companion cell
UDP-G: diphosphate glucose
ADP-G: adenosine diphosphate glucose
CPPU: forchlorfenuron,N-(2-Chloro-4pyridinyl-N-phenylurea)
GA: Gibberellin acid
ip: isopentenyladenine
ABA: Abscisic acid
6-BA: 6-benzylaminopurine
BR: brassinosteroids
EBR: 24-epibrassinolide.
