# Supplementary material for: The Molecular Regulation of Carbon Sink Strength in Grapevine (Vitis vinifera L.)
Source: Front Plant Sci. 2021 Jan 8;11:606918. doi: 10.3389/fpls.2020.606918 (PMC7829256; doi:10.3389/fpls.2020.606918)
Supplement: Supplementary file 1 [file Data_Sheet_1.doc]

Supplementary File S1 The sequence of 19 putative invertase genes identified in the grape genome 12X

Cell Wall Acidic Invertases (cwINV)

>GSVIVT01035389001

MQKLFTLNPYSFKIHSLSFECVVINCHRDGGKSAVKLRKEMGRFGIWVVGLCLMVGGHGIEGETSHHSYRNLQSDPADQPYRTAYHFQPPKNWMNGPMYYNGVYHLFYQYNPYAAVWGNITWAHSTSYDLVNWVHLELAIKPTDPFDINGCWSGSATILTGEEPVIIYTGKDSQNRQVQNLSVPKNISDPLLREWIKSPHNPLMTPIDGIDASNFRDPTTAWQGSDKVWRILSQTPLHSSNKTGMWECPDFYPVSISSRNGVETSVQNAETRHVLKASFNGNDYYIMGKYRRILWAWIQEADKDTEKGWSGLQSFPRSVLLDQNGQRLVQWPVKEIAILHKNQVTFHNKELRGGSVIEVSGITASQADVEVSFDFPHLEEAELMDPSWTDPQALCSRKNVSVKGGIGPFGLLVLASNNLTEQTAIFFRIFKSTQEKHIVLMCSDQSRSSLRQDVDKTIYGAFVDIDLNHEQISLRSLIDHSIVESFGGKGKTCITARVYPELAINTEAHLYAFNSGNQTLNISTLSAWSMKNAEMVPTN

>GSVIVT01035390001

MEISAIWAVGLCLFLARHGIEAEASYPSCRNLQSNPTEQPYRTAYHFQPPKNWMNGPMYYNGVYHLFYQYNPYAAVWGNITWAHSISYDLVNWVHLDHALNPTDPFDINGCWTGSATILPGEEPVIIYTGADTQNRQVQNMAVPKNISDPLLREWIKSPRNPLMSPTNGIDANNFRDPTTAWQGPDKVWRIISQTPLHSSNKTGMWECPDFYPVSTRTGVETSVQNADTQHVLKASFNGNDYYIIGKYRRILWAWIQESDSSSADIEKGWSGLQSIPRSVLLDQTGRQLVQWPIKEIEELRENQVTLLNKEVRGGSVLEVPGITASQVDVEVSFDFPHFKEAEVLDPSWVDPQLLCTQKNASVKGSIGPFGLLVLASKDLTEQTAIFFHIFKTHNKKYVVLMCSDQSRSSVRQDVDKTSYGAFVDIDPLREKISLRGLIDHSIVESFGGEGRSCITARVYPELAINKEAHLYVFNNGTQSVKISRLDAWSMKKAEIVPTNRRRNSHFNQ

>GSVIVT01024570001

MLYHYNFYVAVWGNITWAHSISYVLVKWVNLGHALNPTDPCDINGCWTGSATILPGEEPVIIYIGVDTEIRQFQNRALAKNISDPLHREWMKSPHNPIMTPIDGIDASNFKNPITAWQALLKVWRILDGSLRNGHGTTLFFRSKDFVNWNKSQTLLHSSNKTGMWECANFYSLSNVHKLFTFNPYSSKMCIQ

>GSVIVT01033873001

MKGGSRLLWFLFSLCCYCVIINNNGVEGSHKIHHEYQCVPDTKVRQVHRTGYHFQPPRNWINDPNGPMYYNGIYHLFYQYNPKGAVWGNIVWAHSVSRNLIDWEALEPAIYPSKPFDINGCWSGSATILPGNKPAILYTGIDTQQRQVQNIAFPANLSDPYLRKWVKPDSNPLVVPDVGMNASTFRDPTTAWRVNGHWRMLVGARKKHRGINTGMWECPDFFPVSLYSERGLDTSVTGKNVRHVLKVSLDRTRYEYYTIGRYYPEIDRYIPGNTSADGWSGLRILWGWANESDTADNDTAKGWAGIQTIPRTLWLDKGKKQLLQWPIEELNTLRGQKIQVRNQELKIGENVEITGITAAQADVEVTFSLPSLDKAEEFDPSWVNAQDLCGMKGSTVQGGVGPFGLLTLASEHLEEYTPVFFRIFKAKGKHVVLMCSDAKSSSLRTELYKPSFAGFVDVDLKDGMLSLRSLIDHSVVESFGAGGKTCITSRVYPTLAVSKKAHLYAFNNGTEAVTIKKLNAWTMHRPQMNQ

>GSVIVT01016869001

MATSSHCLLWFFFSLFFGHGFVPLEASHQVYIHLQNQSPSSLKTHQPYRTGYHFQPRKNWMNGIWPMIYKGLYHFFYQYNPHGAVWGNIVWAHSTSTDLVNWTPHKYAISPSQPADINGCWSGSATILPNGKPVILYTGIDPQNKQVQNMAVPKNLSDPFLLEWTKLPQNPLMEPTTINSINASSFRDPTTAWQGTDGRWRVIIGSKIKRKGLAILYRSKDFVRWTKAQHPLHSGKNTGMWECPDFFPVSINSSTGVDTSSISKTLKYVLKLSLDDTKHDYYTIGSYNREKDTYVPDKGSVDNDSGLRYDYGKFYASKTFFDNAKNRRILWGWINESSSVEHDIEKGWSGVQAIPRNVWLDKSGKQLLQWPIAEIEKQRIKPGHMSSRELKGGSKVEVGGITASQADVEISFKISDFKKAEVFDESWSNPQLLCSQRGASVKGGLGPFGLMVLASKGMEEYTAVFFRIFKRQTKYVVLMCSDQSRSSLDNDNDKTTYGAFLDVDPVHEKLSLRSLIDHSIVESFGGGGKVCITARVYPTLAIDGEAHLYAFNKGTGSVGMTTLRAWSMKKAKIN

Vacuolar Acidic Invertases (VIN)

>GSVIVT01001272001 VvVINV1

MVADQWYDTNGVWTGSATLLSDGQVIMLYTGATNESVQVQNLAYPADLSDPLLVDWVKYPGNPVLVPPPGIDDRDFRDPTTAWYWPDGKWRIAIGSKVNKTGISLVYNTEDFKKYELIEGVLHAVPGTGMWECVDLYPVSLKEDNGLDTSFNGPGVKHVLKASLDDDKNDYYAIGTYSLESGNWTPDNSNLDVGIGLRYDYGKFYASKTFYDQNKQRRILWGWIGETDGESADIKKGWASVQSIPRTVVFDKKTGTNILQWPVAEIKSLRKSSKKFDKLEVGPGSVVTLEVEKATQMDITAEFEIDKEALKRIGESDVEYSCGTSGGSAQRGELGPFGLLLLADEGRCEQTPVYFYVAKGTDGQLKTFFCTDESRSSLANDVDKRIFGSTVPVIKGEKLSMRILVDHSIIESFAQGGRTCITSRVYPTKAIYGAAQLYVFNNATSASITASIQTWAMKSAYIRPYSSHQESQ

>GSVIVT01018625001 VvVINV2

MFAWQRTAFHFQPEKNWMNGRNALYPDGPLFHMGWYHLFYQYNPDSAVWGNITWGHAVSRDMIHWLYLPLAMVPDRWFDLNGVWTGSATILPNGQIIMLYTGDTNDSVQVQNLAYPANLSDPLLLHWIKYENNPVMVPPAGIGSDDFRDPTTMWVGADGNWRVAVGSLVNTTGIVLVFQTTNFTDFELLDGELHGVPGTGMWECVDFYPVSINGVYGLDTSAHGPGIKHVLKASMDDNRHDYYALGEYDPMTDTWTPDDPELDVGIGLRLDYGKYYASKTFYDQVKKRRILYGWISEGDIESDDLKKGWASLQSIPRTVLHDNKTGTYLLLWPIEEVESLRTNSTEFEDVLLEPGSVVPLDIGSASQLDIVAEFEVDNETLEAMVEADVIYNCSTSAGAAGRGALGPFGILVLADDTLSELTPIYFYIAKDTDGSYKTFFCTDLSRSSLAVDDVDQRIYGSIVPVLDDEKPTMRVLVDHSIVEGFSQGGRSCITTRVYPTEAIYGAARLFLFNNATGVNVTASIKIWEMASADIHPYPLDQPQ

>GSVIVT01006154001
MLTWQRTGYHFQPEKNWMNDPNGPMFYGGWYHFFYQYNPDAAVWGNIVWGHAVSKDLIEWLHLPLAMVADQWYDTNGVWTGSATLLSDGQVIMLYTGATNESVQVQNLAYPADLSDPLLVDWVKYPVNKTGISLVYNTEDFKKYELIEGVLHAVPGTGMWECVDLYPVSL

Neutral/Alkaline Invertase sub-family

>GSVIVT01024105001

MSELSPKLGQNGTIKNIDSSSTVAETEDIDFSKLSERPRPLTMERQRSYDERNIDSYSRNIDHLDTVFSPCRRSGFNTPRSAMDFEPHPMFAEAWEGLRRSLVFFRGKPVGTIAALDNSDEELNYDQVFVRDFVPSALAFLMNGEPEIVRNFLVKTLRLQSWEKKVDRFQLGEGVMPASFKVLHDPVRNSDTLIADFGESAIGRVAPVDSGFWWIILLRAYTKSTGDSTLAELPECQKGMRLILTLCLSEGFDTFPTLLCADGCCMIDRRMGVYGYPIEIQALFFMALRCALLLLKQDDQGKEFIERIVKRLHALSYHMRSYFWLDMKQLNDIYRYKTEEYSHTAVNKFNVIPDSIPEWIFDFMPTYGGYFIGNVSPARMDFRWFCLGNCVAILSSLATPEQSTAIMDLIESRWEELVGDMPLKVCYPAIEGHEWRIVTGCDPKNTRWSYHNGGSWPVLLWLLTAACIKTGRPQIARRAIELAESRLDPSHLGMISLEEDKQMKPLIKRSASWTF

>GSVIVT01034944001

MNSSSYIGITTMKPYCRVLTSCRNSSIFKFPSSKSNHFIADNSSKFQSKLIHSRRFHCCSAQILGKKCGINSNRRAFRLSDPNWGQIRVYRSCSGAHGGRRGVLVISNVASDFRKHSTSVESHVNEKGFESIYINGGLNVKPLVIERIERGHVEEESGLEFKDPDVNFDHSEGLNKEKVEREVPEIEKEAWRLLRSAVVDYCGNPVGTVAANDPGDKQPLNYDQVFIRDFVPSALAFLLKGEGEIVKNFLLHTLQLQSWEKTVDCYSPGQGLMPASFKVRTVPLDGGNGAFEEVLDPDFGESAIGRVAPVDSGLWWIILLRAYGKITGDYALQERVDVQTGIRLILNLCLTDGFDMFPSLLVTDGSCMIDRRMGIHGHPLEIQALFYSALRCSREMITVNDGTKNLVRAINNRLSALSFHIREYYWVDMKKINEIYRYKTEEYSTDAINKFNIYPDQIPTWLVDWIPDQGGYLIGNLQPAHMDFRFFTLGNLWSIISSLGTAKQNEGILNLIEAKWDDLVAHMPLKICYPALENEEWRIITGSDPKNTPWSYHNGGSWPTLLWQFTLACIKMGRPELARKAVALAEERLSVDHWPEYYDTRNGRFIGKQSRLYQTWTIAGFLTSKMLLENPEMASLLAWEEDYELLEICVCALSKTGRKKCSRSAARSQIPVQ

>GSVIVT01031267001

MITIHCFANSTMKSSSRILLFRRNLPFSGCPLPKSHHFLASNLSNFRINSDHTCKFRSCPLQNLGFRRVIDHTQKFSRVPSPGFGQSRVISSGNVRRLSVISSVSSDVRSFSTSVETRVNDKNFEKIYVQGGMNVKPLVVERIDIDETIENNEESRIESEAEKEAWKLLQDSVVMYCGSPIGTMAANDPGDKTPLNYDQVFIRDFVPSALAFLLKGEGEIVRNFLLHTLQLQSWEKTVDCYSPGQGLMPASFKVRTVPLDGNNEAHEEVLDPDFGESAIGRVAPVDSGLWWIILLRAYGKITGDYTLQERVDVQTGIKLILNLCLTDGFDMFPSLLVTDGSCMIDRRMGIHGHPLEIQALFYSALRCSREMLTQNDASINLVRAINNRLSALSFHIREYYWVDMKKINEIYRYKTEEYSTDATNKFNIYPDQIPSWLMDWVPEEGGYLIGNLQPAHMDFRFFTLGNLWSIISSLGTPKQNQGILDTIQAKWDDLVGHMPLKICYPALEYEEWRIITGSDPKNTPWSYHNGGSWPTLLWQFTLACIKMGRPELARKAVADAEKRLAVDRWPEYYDTRNGRFIGKQSRLFQTWTIAGYLTSKMLLENPEMAALLFWEEDYDLLEICVCGLSKTGRRKCSRFAARSQILVQ

>GSVIVT01031374001

MDLKLGLNSTCSRSTKKSIAEAGILSKLRGFASNGKFAAGGTINDTLGKASIDSIEDEAWNLLRESIVFYCGYPIGTIAANDPSNSSSLNYDQVFIRDFIPSGIAFLLKGEYDIVRSFILHTLQLQSWEKTMDCHSPGQGLMPASFKVRTVPLDGDDSATEDVLDPDFGEAAIGRVAPVDSGLWWIILLRAYGKCSGDLSVQERFDVQTGIKMILKLCLADGFDMFPTLLVTDGSCMIDRRMGIHGHPLEIQALFYSALLCAREMLAPEDGSSALIRALNNRVVALSFHIREYYWIDMRKLNEIYRYKTEEYSYDAVNKFNIYPDQIPPWLVEWMPSKGGYLIGNLQPAHMDFRFFSLGNLWSIVSSLATTDQSHAMLDLIEAKWSELVADMPFKICYPAFEGQEWRITTGSDPKNTPWSYHNGGSWPTLLWQLTVACIKMNRPEIAEKAVKIAEKRISRDKWPEYYDTKQGRFIGKQARLFQTWSIAGYLVSKLLLANPDAANILVNREDSDLVSAFSSMLSANPRRKRDWKGLKQKFIV

>GSVIVT01034753001

MTHRLHGVGGGLYGNTSIHRSQLQSCKCQRADSVSGIASEAGNGTWFVDNAKKRNPINGGSISNGAVETARDTFVKVRVDSIEDEAWDLLRESMVYYCGSPIGTIAAKDPTSSNVLNYDQVFIRDFIPSGIAFLLKGEYDIVRNFILHTLQLQSWEKTMDCHSPGQGLMPASFKVRTVPLDGDDSATEEVLDPDFGEAAIGRVAPVDSGLWWIILLRAYGKCSGDLSVQERIDVQTGIKMILRLCLADGFDMFPTLLVTDGSCMIDRRMGIHGHPLEIQDFDQMKSSL

>GSVIVT01034754001

MGGWLTLLSSDTIEALFYSALLCAREMLAPEDGSADLIRALNNRLVALSFHIREYYWIDMKKLNEIYRYKTEEYSYDAVNKFNIYPDQISPWLVEWMPNKGGYLIGNLQPAHMDFRFFSLGNLWSIISSLATMDQSHAILDLVEAKWGDLVADMPLKICYPALEGQEWQIITGSDPKNTPWSYHNAGSWPTLLWQLTVACIKMDRPQIAAKAVEIAERRIARDKWPEYYDTKKARFIGKQACLFQTWSIAGYLVAKLLLSDPTAAKILITEEDSELVNAFSCMISANPRRKRGRKSSTQTFIV

>GSVIVT01009904001

MSPIPMDVYSNGNVKNLETASTTVQIDDSDFLRLLDRPRPISIERNRSFEEKSFNELSSTLSPLLFHRNVEKNSFHIFDLLDHTFSPVRSSLNTPRSNHCFEPHPVFTDAWEALRRSLVYFRGQPVGTIAAIDHSSDELNYDQVFVRDFVPSALAFLMNGEPEIVKNFILKTLRLQSWEKKVDQFKLGEGVMPASFKVFHDPVRNYETLIADFGESAIGRVAPVDSGFWWIILLRAYTKSTGDSSLAEMPECQRGMRLILSLCLSEGFDTYPTLLCADGCCMIDRRMGVYGYPIEIQALFFMALRCALLLLKQDDKGKEFVELISKRLHALSYHMQSYFWLDIKQLNDIYRYKTEEYSHTAVNKFNVMPDSLPDWVFDFMPSRGGYFIGNVSPAKMDFRWFCLGNCVAILSSLATPEQSSAIMDLIESRWQELVGEMPLKICYPAFESHEWRIVTGCDPKNTRWSYHNGGSWPVLIWLLTAACIKTGRPQIARRAIELAESRLLKDNWPEYYDGKLGRYIGKQARKFQTWSIAGYLVAKMMLDDPSHLGMISLEEDKQLKPLFKRSLSWSH

>GSVIVT01034488001

MYSPGGRSGFDTPASSARNSFEPHPMVNEAWEALRRSLVFFRGQPVGTIAAYDHASEEVLNYDQVFVRDFVPSALAFLMNGEPEIVKNFLLKTLHLQGWEKRIDRFKLGEGAMPASFKVLHDPIRKTDTLIADFGESAIGRVAPVDSGFWWIILLRAYTKSTGDLSLAETPECQKGMKLILTLCLSEGFDTFPTLLCADGCSMVDRRMGIYGYPIEIQALFFMALRCALAMLKQDSEGKECIERIVKRLHALSYHMRSYFWLDFQQLNDIYRYKTEEYSHTAVNKFNVIPDSIPEWVFDFMPTRGGYFIGNVSPARMDFRWFALGNCVAILSSLATPEQSMAIMDLIESRWEELVGEMPLKISYPAFENHEWRIITGCDPKNTRWSYHNGGSWPVLLWLLTAACIKTGRPQIARRAIDLAESRLLKDSWPEYYDGKLGRYVGKQARKYQTWSIAGYLVAKMLLEDPSHLGMISLEEDRQMKPLIKRSSSWTC

>GSVIVT01027176001

MPEKKPTSSDSASTLDAKKLNLLPAVKYLLKELDLEHVDLDNLPQMVEAESYSYPDVTETSEKPLDSPAEASGEKNEQSTENVSGGSGRSVPTLKESHSVGASLERYDQIREDKGVKKTDEPEKPLVFRSEVNEEKVVCTLKQPLNVETNIGNLDQNTSPGSRLSGHQVTGTEGASLVDEAWDRLQKSFVYFRGKPVGTLAAIDPSAEPLNYNQVFVRDFVPSGLACLMKNPPEPEIVKNFLLQTLHLQGCQKKIDNYTLGEGVMPASFKVLHDPKTQKETLVADFGGSAIGRVAPVDSVFWWIILLRSYTKCTGDNSFSELPQVQGGIKSILKLCLCDGFNNFPTLLCADGCCMVDRSMGINGYPIEIQSLFYFALRCARQMLKPEHGGKEFFKRIDARITALSFHVQTYYWLDITQLNNIYRYKTEEYSHTAVNKFNIIPDSIPEWLFDFMPLRGGYFMGNVSPGRMDFRWFLAGNCIAILSSLATSEQATAIMDLVEERWEQLIGEVPLKVVYPALEGHYWELVTGCDPKNTPWSYHNGGSWPVLLWLLTAACIKIGRPQIAKRAIDLVEQRLSKDGWPEYYDGKTGRYVGKQARKFQTWSIAGYLVAKMMIENESNLLVISHEEEKKTNKLRHTRSASCFFQ

>GSVIVT01037430001

MKGELEIVKNFLLRTLHLQLSVKGIDRFALGQGLMGVDTLIADFGETAIGRVAGVDSGFWWIILLHAYTRATGDYSLSHRPECQNGMKLILSVCLAEGFDTFPTLLCADGCGMADRRMGVYGYPIEIQALFFMALRCAVHLLQEDDGKEFIMRIEKRLQALTYHMRSYFWLDFQQLNNIYRYKTEEYSHTAVNKFNVIPDSIPDWVFDFMPMKGGYFIANVSPARMDFRWFVLGNCVAILSSLATHNQSMAILDLIEERWGELVGKMPLKLSYPALDIHGWSIETGSDPKNTRWSYHNGGSWPGLLWLVTAACIKTGRPEIARKAIELAEQRLSKDDWQEYYDGKEGCYVGKQSRRLQTCSIAGYLVSKMLLEEPSHLGIIALEEDEKIKPTTITRSTTLPTKFRGYP

>GSVIVT01037429001

MYLKLFLKNNYFCIKFIKDCQPASNFFILQHAYTRATGDYSLSHRLECHNGMKLILSVCLVEGFGTFPTLLCADGCCVTDRRMGVSGYPMEIQALFFMALRCAVHLLREDDGKEFSKRIEKRLQALTYHMRSYFWLDFQQLNNIYRYKTEEYSHTAVNKFNVMPNSIPDWVFDFMPMKGGYSVANVSPTRMDFRWFVLGNCVAILSSLATYNQSMAILDLIEDRWEELVGKMPLKLSYPALDIHGWSIETGSDPKNTRWSSQNGGSWPGLLWLLTAACIKTGWPEIERKAIELAEHAADVQGRLARIL
